# Detecting Inspection Objects of Power Line from Cable Inspection Robot LiDAR Data

**DOI:** 10.3390/s18041284

**Published:** 2018-04-22

**Authors:** Xinyan Qin, Gongping Wu, Jin Lei, Fei Fan, Xuhui Ye

**Affiliations:** 1 Department of Power and Mechanical Engineering, Wuhan University, Wuhan 430072, China; xyqin@whu.edu.cn (X.Q.); fei-fan@whu.edu.cn (F.F.); xhye@whu.edu.cn (X.Y.); 2 Key Laboratory of Hydraulic Machinery Transients, Wuhan University, Ministry of Education, Wuhan 430072, China

**Keywords:** cable inspection robot, LiDAR, detection, recognition, power line, inspection object

## Abstract

Power lines are extending to complex environments (e.g., lakes and forests), and the distribution of power lines in a tower is becoming complicated (e.g., multi-loop and multi-bundle). Additionally, power line inspection is becoming heavier and more difficult. Advanced LiDAR technology is increasingly being used to solve these difficulties. Based on precise cable inspection robot (CIR) LiDAR data and the distinctive position and orientation system (POS) data, we propose a novel methodology to detect inspection objects surrounding power lines. The proposed method mainly includes four steps: firstly, the original point cloud is divided into single-span data as a processing unit; secondly, the optimal elevation threshold is constructed to remove ground points without the existing filtering algorithm, improving data processing efficiency and extraction accuracy; thirdly, a single power line and its surrounding data can be respectively extracted by a structured partition based on a POS data (SPPD) algorithm from “layer” to “block” according to power line distribution; finally, a partition recognition method is proposed based on the distribution characteristics of inspection objects, highlighting the feature information and improving the recognition effect. The local neighborhood statistics and the 3D region growing method are used to recognize different inspection objects surrounding power lines in a partition. Three datasets were collected by two CIR LIDAR systems in our study. The experimental results demonstrate that an average 90.6% accuracy and average 98.2% precision at the point cloud level can be achieved. The successful extraction indicates that the proposed method is feasible and promising. Our study can be used to obtain precise dimensions of fittings for modeling, as well as automatic detection and location of security risks, so as to improve the intelligence level of power line inspection.

## 1. Introduction

**Figure 1:**
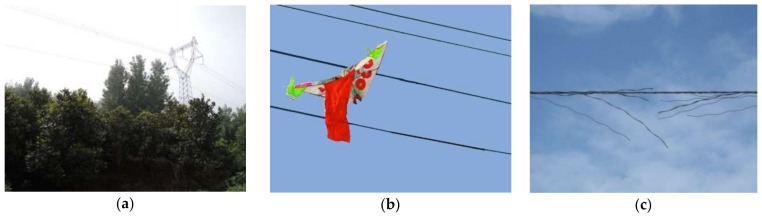
The hidden troubles in safe operation of power line: (**a**) Vegetation encroachment; (**b**) Attachment; (**c**) Broken strand.

Power lines are important parts of the national power grid. With the development of the economy, power towers and power lines are rapidly increasing. The safe operation of a power line is facing many challenges, as shown in Figure 1. Firstly, power lines are extending into dense forests. The long branches could cause line tripping, and at the same time, people pay more attention to the protection of vegetation, further exacerbating the contradiction of trees and power lines exhibited in Figure 1a. Secondly, the impact of human activities is increasing, especially in the vicinity of urban areas, and attachment objects such as kites and balloons are frequently attached to power lines that can cause security risks, as shown in Figure 1b [1]. Thirdly, power lines exposed in the wild suffer from mechanical tension and electrical flashover, leading to the problem of broken strands, as shown in Figure 1c. Therefore, it is very important to regularly inspect power lines to ensure their stable operation [2].

The traditional patrolling method is time-consuming, laborious, and has a low positioning accuracy, making it difficult to meet the requirements [3]. With the rapid development of detection technology, the use of a LiDAR measurement system is a good way to solve these problems [4]. As a result, airborne LiDAR and vehicle-borne LiDAR are increasingly used in power line inspection. From the perspective of the current application situation, airborne LiDAR is not subject to geographic factors and can collect large-scale spatial data relating to power lines. So, it has been obviously effective in terms of power line modeling and safe distance measurement [5]. However, airborne LiDAR is highly restricted by high-speed flying and long-distance scanning, resulting in large point cloud spots and sparse point clouds. The point clouds of airborne LiDAR can describe the approximate shape of power lines, but cannot obtain the precise data of inspection objects on the power lines or their surrounding areas [6]. Vehicle-borne LiDAR can collect finer point clouds of power lines at close range [7]. However, vehicle-borne LiDAR cannot run across mountains along power lines, mainly used in parts of urban areas [8]. The ability to combine the collection ability of airborne LiDAR and collection quality of vehicle-borne LiDAR requires obtaining high-quality point clouds of power lines, which is the source of research in this paper to expand the current inspection content of LiDAR in the field of power line inspection.

The cable inspection robot (CIR), a special robot, is used for the inspection of power lines [9,10]. The State Grid has implemented the automatic inspection demonstration application of CIR across primeval forests [11]. CIR has gradually matured, possibly providing a new carrying application of LiDAR. The main contribution of this paper is to construct the segmentation strategy from the layer to the block based on prior information and position and orientation system (POS) data, i.e., the walking trajectory of CIR, and to propose a complete method of detecting various inspection targets of power lines. The proposed method can automatically detect these small-size inspection objects on the power line, such as dampers, and obtain their geometrical models and precise locations. The recognition of these inspection objects is a good complement to the current spatial model of the point cloud of the transmission corridor, thus establishing a complete 3D model of the transmission corridor. Based on the 3D model, inspectors can guide power robots to accurately locate these abnormal points to diagnose or maintain, effectively improving the intelligent level of power line inspection.

## 2. Literature Review

The inspection objects extracted from LiDAR data mainly involve three aspect works, i.e., ground point removal, power line extraction, and inspection object recognition. The literature review elaborates on these three aspects, respectively.

Ground point removal from the original point cloud, retaining these waiting-classification points, is called filtering [12]. Filtering directly determines the quality of subsequent point cloud processing. Many scholars have studied the filtering methods of laser point cloud data, which are roughly divided into two categories: the partial extension method and interpolation method. The former method refers to local to a wide range of terrain, including the morphology filtering method [13,14] and the terrain slope filtering method [15]. The latter method’s scale is from global gradually refined to local, and the typical methods are the linear prediction method proposed by Kraus et al. [16,17] and the Triangular Irregular Networks (TIN) proposed by Axelsson [18]. The interpolation method is more extensive due to its practical application. The essence of interpolation is a densified process in an iterative process, which is greatly influenced by the initial digital elevation model (DEM), and there is an error accumulation. Therefore, in mountainous areas with great fluctuation, the parameter of the algorithm needs to be carefully considered before filtering. In addition, ISPRS Working Group III/3 [19] conducted a test to determine the performance of the eight filtering algorithms, both qualitatively and quantitatively. Their conclusion was that all filtering algorithms would produce errors in complex urban areas and rough terrain with vegetation. In sum, the filtering algorithm is time-consuming and usually takes up about 60–80% of the laser point cloud data processing time and computing resources [20].

Many researches have focused on power line extraction from airborne laser scanning (ALS) data. Melzer and Brieser [21] extracted power line points using a bottom-up strategy and applied the 2D Hough transform and RANSAC algorithm to identify power lines. McLaughlin [22] classified power line points based on their point features using a Gaussian mixture model for distinguishing power lines from their surroundings. Jwa et al. [23] proposed a voxel-based piecewise line detector (VPLD) algorithm to extract power line points by a comprehensive approach combining the Hough transform, feature eigenvectors, and point density. Zhu and Hyyppa [24] firstly identified power line candidate points by statistical analysis considering height, point density, and histogram thresholds, which were then converted to 2D images with the image processing method to extract power lines from ALS data. However, the experimental results show that the point distance of ALS data will widen with increased flight height [25], for example, in reference [26], the point clouds were obtained at a height of 400 m, so the point distance reached 1 m. Therefore, better extraction results cannot be obtained due to a higher flight height. To obtain a high-quality power line point cloud, there have been some studies of power line extraction based on mobile laser scanning (MLS) data. Ou et al. [27] made an attempt to extract a power line point cloud based on a height constraint and the Hough transform from MLS data. Cheng et al. [28] firstly proposed a voxel-based hierarchical method to extract power line points from MLS data. Then, a bottom-up method and clustering recovery procedures were used to identify each power line. There are two limitations to power line extraction from MLS data: one is that many thresholds are specified by experience; and the other is the frequent occlusions by trees or buildings. Power line points of MLS present a dense breakage-dense distribution pattern, greatly influencing the extraction accuracy.

There are very few reports on inspection object recognition of power lines, because of the difficulty in obtaining high-quality point clouds. Therefore, a review of studies that propose specific approaches of recognizing components of electrical substations from LiDAR data is provided for sharing similarities with inspection objects on the power line. Xu et al. [29] introduced typical LiDAR applications in the electrical power industry by employing LiDAR data, such as power lines and electrical substations. They pointed out the most important work for power line checking was to find the abnormal equipment, dangerous points, and the threat to lines. However, these works are limited by the distance between lines and buildings, and whether vegetation was suitable for the safety requirement. Arastounia and Lichti [30] proposed a knowledge-based segmentation method finding the principle direction of points’ distribution to recognize points on insulators from a subset of an electrical substation point cloud. Then, they [31] improved an automated methodology to recognize six key components of electrical substations. In this article, they indicated that, at present, no comprehensive automated solution has been proposed to recognize all components of electrical substations from LiDAR data due to the complexity of the parts. The case is similar for recognizing inspection objects of power lines. Therefore, this is followed by a review of the types of geometric primitive methods for segmentation and object recognition from LiDAR data. Sohn et al. [32] employed a Markov random field (MRF) classifier, which emphasized the roles of the spatial context of linear and planar features as in a graphical model. Belton and Lichti [33] proposed a segmentation algorithm through local covariance analysis to segment smooth surfaces, edges, and boundaries in an industrial site comprising planar and cylindrical surfaces. Pu and Vosselman [34] segmented planar surfaces using a surface-growing algorithm, which recognized various objects by semantic features. Zhu et al. [35] segmented planar surfaces using PCA and eigenvalues analysis.

This paper is organized as follows. Section 3 outlines the CIR LiDAR system, and analyzes the new characteristics of CIR LiDAR data. Section 4 details the novel inspection object extraction method. Section 5 illustrates the three datasets to verify the effectiveness and feasibility of the proposed method. Section 6 expounds the results of all datasets and provides the corresponding discussions. Conclusions are drawn in the last section.

## 3. Materials and Data Analysis

### 3.1. CIR LiDAR

CIR LiDAR is a novel point cloud collection mode using CIR as a motion platform. The work of CIR LiDAR is similar to airborne/vehicle-borne LiDAR, which aims to collect scene spatial information through integrating laser scanning (LS), global positioning (GNSS), and inertial navigation (INS).

**Figure 2:**
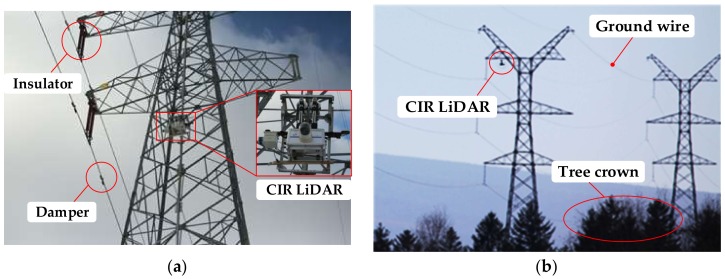
The image of collection data of CIR LiDAR system: (**a**) close-up image; (**b**) long-distance image.

CIR LiDAR is specifically designed for scanning the transmission corridor along power lines. Figure 2a,b are images of CIR LiDAR, where CIR LiDAR is sent to the walking ground wire by the automatic up-down-line device. CIR LiDAR can scan these inspection objects in close-range, including the insulator, damper, ground wire, attachment (e.g., kite and balloon), tree crown, crossing power line, and so on.

Detecting these inspection objects has three main difficulties. First, size differences among these objects are very large. For example, the inspection object (damper) on the power line is almost invisible in Figure 2. Second, in order to save land, the layers and lines of power lines are increasing, and the distribution of power lines is increasingly complex. Third, because power lines are long, the amount of power line LiDAR data is very large, and the scene is monotonous and very similar. Solving these difficulties for the successful detection of various inspection objects is the main aim of this paper.

### 3.2. Data Analysis

**Figure 3:**
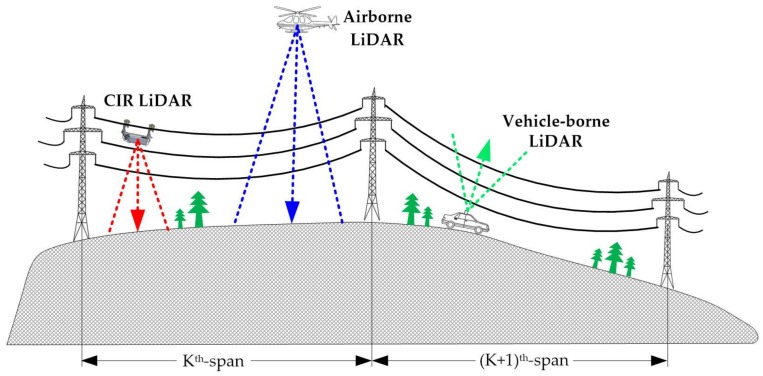
Schematic diagrams of work principles of three LiDAR systems.

CIR LiDAR is integrated on the special robot, and the ground wire is used as its moving path to closely scan the transmission corridor, as shown in Figure 3. Its scanning perspective is different from both airborne top-down scanning and vehicle-borne bottom-up scanning. So, the CIR LiDAR data have many distinctive characteristics.

Firstly, CIR LiDAR focuses on transmission corridors that belong to the structured working condition. Towers and loops must strictly comply with the power erection standard, so power line point clouds present the following characteristics:

■ A whole line can be divided into many span segments with a similar power line structure.
■ Power lines are usually higher than other objects in a span segment.
■ Power lines in a span segment are parallel and extensible, and they present different spacing in different layers.
■ Power lines belong to the natural suspension line following the hyperbolic cosine equation.

Secondly, CIR POS data can be used not only for generating point clouds, but also for processing point clouds. CIR POS data could represent orientations and shapes of all power lines in the same span segment because CIR moves along the power line. Meanwhile, the CIR POS data derived from different data sources is naturally separated from point cloud datasets.

Finally, CIR LiDAR scans transmission corridors at a low speed (4 km/h). The point clouds are very dense, and can thus obtain precise spatial data of various fittings on power lines and abnormal points around power lines. Based on the distinctive CIR LiDAR data, a new method is proposed in this paper to automatically recognize inspection objects surrounding power lines.

## 4. Methodology

**Figure 4:**
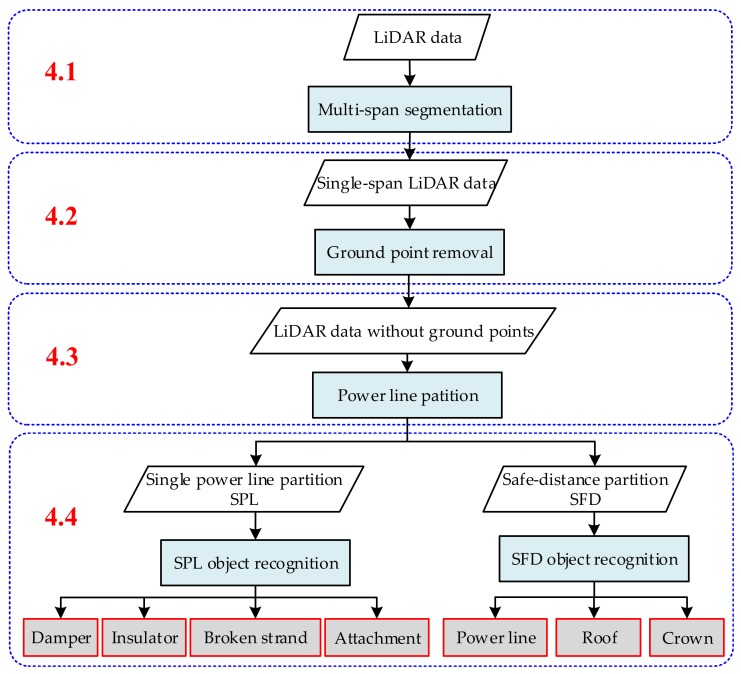
Flowchart of the entire methodology.

The methodology is described in the following four subsections, as shown in Figure 4. In Section 4.1, the original point cloud collected by CIR LiDAR is divided into single-span point cloud datasets. In Section 4.2, a large number of ground points are removed in a single-span point cloud dataset, while the remaining points are retained with close-relationship power line points. In Section 4.3, the remaining point cloud is divided into two partitions, i.e., single power line (SPL) partition and safe distance (SFD) partition, which permits us to better classify and recognize inspection objects in different partitions. In the last section, the abnormal point clouds in different partitions are identified as the specific inspection objects.

### 4.1. Multi-Span Segmentation

The original point cloud collected by CIR LiDAR usually includes multi-span data. For the same transmission line, the distributions and trends of power lines are basically identical for the multi-span segment. In addition, CIR POS data to generate point clouds can reflect geographic information. In our study, a span segment dataset is regarded as our data processing object. We mainly focus on detecting inspection objects surrounding power lines, so the single-span dataset does not contain the tower data. In order to ensure the safe operation of the robot on the ground wire, a variety of position sensors are installed on the body of CIR. The fusion of position data derived CIR position sensors and LiDAR data is undertaken to generate the high-precision CIR POS. When the robot passes through different span segments, it presents the obvious uphill-downhill process. By analyzing the symbol change of the angle data measured by the angle sensors, the center points can be defined as the suspension points to segment multi-span data so that the original point cloud is divided into single-span datasets.

### 4.2. Ground Point Removal

Although the original point cloud is divided into single-span datasets, the dataset of a span still has a very large number of points due to CIR scanning at a low speed. It provides the possibility for object recognition and also causes the difficulty of data processing. Therefore, a large number of ground points need to be removed to free up computing resources. We do not use the existing filtering algorithms, but make full use of the strict power line construction specification and the elevation of CIR POS to construct the optimal elevation threshold defined as Equation (1):

(1) $$Z_{opt}=Z_{\mathrm{POSmin}}-\sum_{1}^{k}H_{i}-d,$$

where $Z_{opt}$ is the optimal elevation threshold; $Z_{\mathrm{POSmin}}$ is the lowest height of POS in the single-span segment; and $H_{i}$ is the vertical distribution matrix for all power lines in single-span segment. The erection specification of power lines can provide accurate prior data. *d* is the adjusted value specified by the specific situation of the power line and inspection requirement. Therefore, these points, corresponding to their Z-coordinate less than the $Z_{opt}$, belong to ground points that will be eliminated from the single-span point cloud. At the same time, these points closely related to power lines are also well preserved.

### 4.3. Power Line Partition

This section will further partition the single-span point cloud after ground point removal. The aim of power line partition includes three aspects: First, the inspection objects are different in different areas of the power line, so different algorithms can be used to identify them to reduce the difficulty of recognition; second, the sizes of inspection objects greatly change in different areas of the power line, and the partition is convenient to set the appropriate thresholds to improve the accuracy of recognition; and third, many inspection objects distributed around power lines are small, so are difficult to identify. Power line partition can highlight the characteristics of the point cloud of the inspection object and reduce the interferences of other points to ensure the accuracy of recognition. The partition strategy aims to divide from “layer” to “block” according to the distributions of power lines and inspection requirements. The model of the power line is catenary, which cannot simply partition through the elevation. Therefore, we propose a structured partition based on a POS data (SPPD) algorithm that reflects the characteristics of power lines in a span segment.

**Figure 5:**
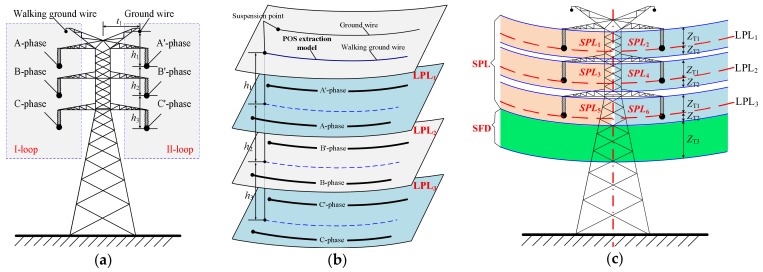
The schematic of power line partition: (**a**) erection specification of power line; (**b**) partition at the layer-level; (**c**) partition at the block-level.

In this paper, a typical double-loop transmission line is taken as an example to illustrate the specific process of the SPPD algorithm from “layer” to “block”. There are two ground wires and six power lines (in this paper, power lines do not consider as multi-bundle wires). The erection specification of power lines is shown in Figure 5a.

First, partition at the layer-level is undertaken, as illustrated in Figure 5b. The six power lines are divided into three layers by the erection specification of the power line. The positions of the three layers, i.e., LPL_1_, LPL_2_, and LPL_3_, can be determined based on the POS extraction model and the clearances (*h*_1_, *h*_2_, and *h*_3_). The LPL_3_, the lowest layer, is also labeled as LPL_last_.

Second, partition at the block-level is completed, as illustrated in Figure 5c. The LPL_1_ surface is upward shifted *Z*_T1_ (*Z*_T1_ = L, L is the length of the insulator) as the upper boundary, and downward shifted *Z*_T2_ (*Z*_T1_ = L/2) as the lower boundary. The block area enclosed by these two surfaces includes two power lines. The center plane of the tower is used to split the block area into two sub-partitions (i.e., *SPL*_1_ and *SPL*_2_). For the LPL_2_ and LPL_3_ layers, the *SPL*_3_–*SPL*_6_ partitions can be respectively obtained by the above process. For the lowest layer LPL_last_, the LPL_last_ surface is downward shifted *Z*_T2_ as the upper boundary, and downward shifted *Z*_T3_ as the lower boundary. The block area enclosed by the surfaces is defined as the SFD partition.

Finally, these power lines are divided into six SPL partitions and one SFD partition. In each SPL partition, there is only one power line and its surrounding point cloud, which mainly detects fittings and abnormal points closely related to power lines. In SFD partition, including the point clouds under the lowest power line, this partition mainly detects the hidden dangers below the lowest power line.

In this paper, the steps of the SPPD algorithm [36] are listed as follows:

(1) The 3D point cloud projected to the vertical plane of POS is used to generate a 2D point cloud;
(2) The extraction model is built up by fitting the POS data on the vertical plane;
(3) Due to the additional sag resulting from the self-weight of CIR, the extraction model is modified with the additional sag, as follows:
  (2) $$\begin{array}{l} z=Ax^{2}+Bx+C+\delta (x)\\ \delta (x)=\frac{\eta x(L-x)W}{\sigma_{0}LS} \end{array},$$

where δ(x) is the additional sag; *S* is the cross-sectional area of ground wire; *W* is the weight of CIR; *L* is the horizontal spacing; $\sigma_{0}$ is the horizontal stress of the power line; and η is the increased coefficient of the impact effect. These parameters are stored in the CIR inspection database.

(4) The point clouds of SPL and SFD partitions are extracted by the modified extraction model and the parameters (*Z*_T1_, *Z*_T2_, *Z*_T3_);
(5) The 2D point cloud is mapped back to three-dimensional space to obtain the 3D point clouds of two kinds of partitions.

### 4.4. Inspection Object Recognition

The different types of inspection objects have their own characteristics [37], as illustrated in Table 1. The abnormal points can be recognized through distribution features of point clouds and geometric characteristics of objects.

According to the feature description in Table 1, there are regular distribution areas of various inspection objects. Therefore, the two algorithms are used to recognize objects in SPL and SFD partitions, respectively.

#### 4.4.1. Recognition in SPL Partition

**Figure 6:**
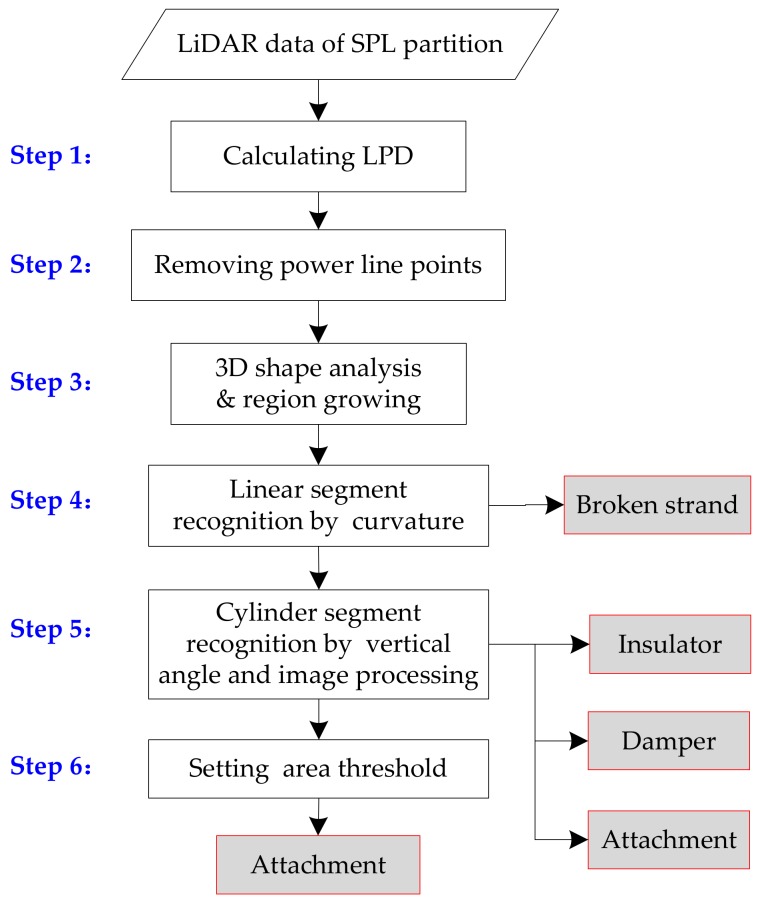
Flowchart of SPL partition recognition.

In the SPL partition, the inspection objects mainly include a damper, insulator, broken strand, and attachment. The algorithm flow on the recognition of these objects is shown as Figure 6.

The specific procedures are listed as follows.

Step 1 Step 1 Calculate local point density (LPD) of each point in the dataset **S** defined as [38]:
  (3) $$\begin{array}{l} S={\left\{p_{i}\right\}}_{i=1}^{N}\\ D_{i}=\frac{k}{\pi {d^{2}}_{k}}\begin{array}{c} \left(i=1,\cdots ,N\right) \end{array} \end{array},$$

where **S** is point set of the SPL partition; *D_i_* is the local point cloud density of the point *p*_i_; *k* is the number of the nearest neighbor points of the reminded point; and *d_k_* is the distance between the reminded point and the farthest point in the *k*-nearest neighbor.

Step 2 The abnormal point cloud is denser than the point cloud of the power line in SPL partition. In addition, the point cloud of the power line is not only large in quantity, but also connects to these abnormal point clouds. It is not convenient to detect inspection objects, especially for small-size inspection objects. Therefore, this step is employed to remove power line points or sparse points based on the LPD characteristics, only retaining abnormal point blocks with an independent distribution. The threshold of removing power line points is determined from the density histogram of the entire point cloud. The dominant peak corresponding to power line points is automatically identified, from which LPD is determined. These points on the power line or their densities below the power line are then easily removed, and the remaining points are retained for the subsequent processing.
Step 3 We use principle component analysis (PCA) to analyze the spatial distributions of neighborhoods of points. The optimal neighborhood radius of each point is determined by the neighborhood adaptive method [39]. The PCA generates three eigenvalues: *λ*_max_, *λ*_mid_, and *λ*_min_ (*λ*_max_ ≥ *λ*_mid_ ≥ *λ*_min_ ≥ 0), and three eigenvectors: ${\vec{e}}_{\max}$, ${\vec{e}}_{\mathrm{mid}}$, and ${\vec{e}}_{\min}$. Each eigenvalue (*λ_i_*) represents the dispersion of a neighborhood in the direction of its corresponding eigenvector (${\vec{e}}_{i}$). The eigenvalues are normalized with Equation (4) to generate new eigenvectors *λ_n_*_max_, *λ_n_*_mid_, and *λ_n_*_min_.
  (4) $$\lambda_{ni}=\frac{\lambda_{i}}{\lambda_{\max}+\lambda_{\mathrm{mid}}+\lambda_{\min}},i=\max,\mathrm{mid},n\min,$$

We assume that points corresponding to the un-removed power line and broken strand are a subset of the linear points, and that points corresponding to the damper and insulator are a subset of the cylindrical points. We define a linear point as a point with *λ_n_*_max_ = 1 and *λ_n_*_mid_ = 0, and a cylindrical point as a point with *λ_n_*_max_ > 0.5 and *λ_n_*_mid_ = *λ_n_*_min_.

The 3D region growing is employed. A point is first chosen at random (the seed), and a new empty component is created. The region growing segments of these points is identified as belonging to the shaped neighborhood. We have empirically verified that it is suitable to set up the threshold of 3D Euclidean distance as 0.15 m. Then, we divide the LiDAR returns into three disjoint subsets: “linear” points, “cylindrical” points, and remaining “block” points.

Step 4 The un-removed power lines and broken strand in Step 2 are two types of linear objects that appear in LiDAR data of the SPL partition. The power line appears as a straight linear object, while the broken strand appears as a curvilinear object. Therefore, they can be distinguished on the basis of their curvature, and the standard deviations of the object’s principal direction vectors (${\vec{e}}_{\max}$) in three dimensions, i.e., ($\sigma_{e_{x}},\sigma_{e_{y}},\sigma_{e_{z}}$). If $\sigma_{e_{x}}=\sigma_{e_{x}}=\sigma_{e_{x}}=0$, it means that the linear object is the power line point; if the linear object has at least one non-zero standard deviation along one of the three cardinal directions, it reveals that the linear object is the broken strand.
Step 5 The damper and insulator are two types of cylindrical objects in SPL partition. In this paper, the damper is parallel to the horizontal installation, and the insulator is perpendicular to the horizontal installation and has a certain length. So, they can be recognized by the vertical angle $\theta_{va}$, i.e., the angle between the object’s principal direction vectors (${\vec{e}}_{\max}$) and the vertical direction, calculated by $\theta_{va}=\arctan(\sqrt{e_{x}^{2}+e_{y}^{2}}/e_{z})$. These cylindrical objects ($\theta_{va}$ < $45^{\circ }$) are further identified with the elevation difference ΔZ. If the value is greater than ΔZ, the object is the insulator; otherwise, it is the attachment. These cylindrical objects ($\theta_{va}$ > $45^{\circ }$) will be further identified with the image processing method.

These remaining points are projected in the front view, and are resampled to generate a grayscale image. By binarization, the grayscale image is used to generate the binary image *I_bo_*. Due to the inhomogeneity of point cloud distribution, there are many holes in the binary image. The binary image *I_b_* is filled using a morphological closed-operation [40]. The formula is as follows:

(5) $$I_{b}=I_{bo}\cdot B=(I_{bo}⊕B)\Theta B,$$

where *B* is the structural element; “⊕” represents the dilation operation; and “Θ” represents the erosion operation.

Then, shape matching pretreatment of *I_bo_* is completed. The edge detection is performed using a Canny operator. Then, the shape contour is sorted and marked by means of connected tracking, and the contour points of the object are obtained.

Following this, objects using the shape context (SC) algorithm are identified [41,42]. Firstly, contour feature points are uniformly sampled along the boundary. Secondly, the inner distance shape context feature of the contour feature points is extracted to express the form of the shape statistics histogram.

(6) $$C_{ij}=\frac{1}{2}\sum_{1}^{N}\frac{{\left[h_{P,i}(k)-h_{Q,j}(k)\right]}^{2}}{h_{P,i}(k)+h_{Q,j}(k)},$$

where $h_{P,i}(k)$ is the shape statistics histogram of point *p_i_* of object P; $h_{Q,j}(k)$ is the shape statistics histogram of point *q_i_* of object model Q; and *N* is the statistic value of the histogram (bins). Thirdly, using the dynamic programming matching algorithm [43], the optimal solution is obtained by Equation (6), and the matching cost of the whole shape is obtained to identify the damper; otherwise, it is the attachment.

(7) $$H(\pi )=\sum_{i}C(p_{i},q_{\pi (i)}),$$

where π is the replacement match, where the sum of matching costs of all points is minimized. Next, the obtained data sets of each object back to 3D space are mapped, and we get the point cloud blocks of the corresponding inspection objects.

Step 6 For the remaining “block” points, calculate the projection area to the XY-plane, where the point clouds greater than the given threshold are considered as the attachment.

#### 4.4.2. Recognition in SFD Partition

**Figure 7:**
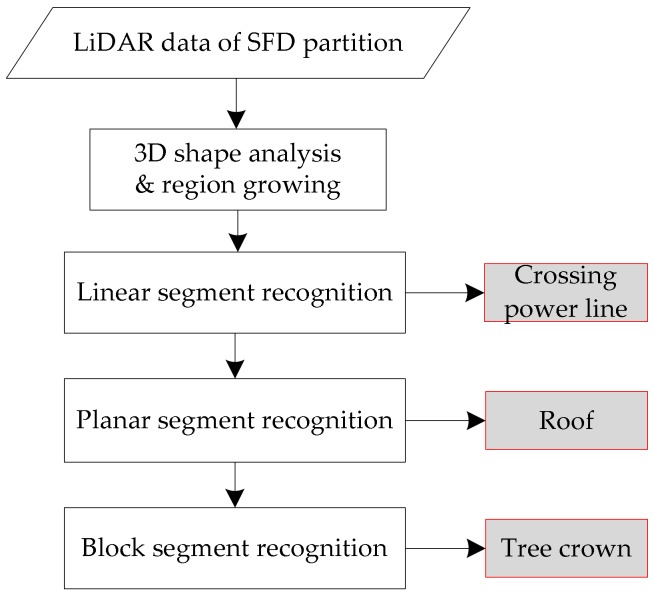
Flowchart of SFD partition recognition.

In SFD partition, the inspection objects are relatively simple, mainly including the tree crown, roof, and crossing power line. These objects have large shape sizes. The algorithm flow is used to recognize these objects, as shown in Figure 7.

The PCA method is used to analyze the spatial distributions of neighborhoods of points. We assume that points corresponding to the crossing power line are a subset of the linear points, and that points corresponding to the roof are a subset of the planar points. Based on the interpretation of PCA as described above, we define a linear point as a point with *λ_n_*_max_ = 1 and *λ_n_*_mid_ = 0, and a planar point as a point with *λ_n_*_max_ = *λ_n_*_mid_, and *λ_n_*_min_ = 0.

The region growing segments of the points is identified as belonging to the linear neighborhood by the threshold of 3D Euclidean distance (2 m), and the cosine of the angle between the principal directions of the neighboring points is greater than the threshold (10°). The region growing segments of the points is identified as belonging to the planar neighborhood by the threshold of 3D Euclidean distance (2 m), and the cosine of the angle between the normal vectors of the neighboring points is greater than the threshold (10°). The region growing segments of the remaining points is the threshold of 3D Euclidean distance (3 m). Then, we divide the LiDAR returns into three disjoint subsets: “linear” points (crossing power line); “planar” points (roof); and remaining “block” points (tree crown).

## 5. Datasets

In this paper, there were three datasets collected by two CIR LiDAR systems. Among them, the first dataset and the second dataset were collected from the same testing site, and they were used to verify the effectiveness of the proposed method in SFD and SPL partition, respectively. The third dataset was collected from the actual line, showing the feasibility of the proposed method.

### 5.1. First Dataset

**Figure 8:**
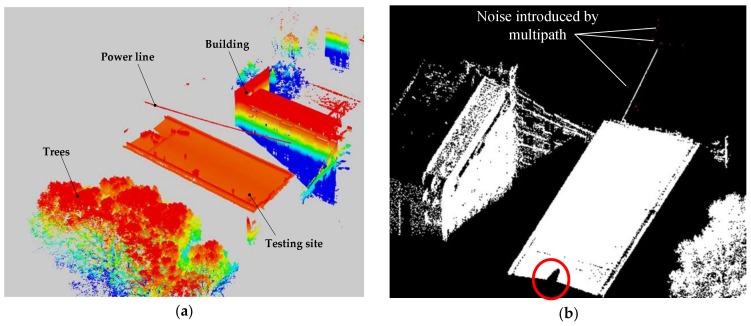
Point cloud of the first dataset (**a**); Multipath-introduced noise and black hole in the dataset (**b**).

The first dataset was captured from a testing site which is 50 m in length and 30 m in width, located on the top floor of a building. The experiment was specifically designed to verify the recognition method for SFD partition. So, two parallel power lines without any fittings were placed at the testing site. There are some tall trees and buildings around the testing site. CIR moves along the ground wire at a speed of 4 km/h to collect data. Figure 8a is the color-coded first dataset by elevation in which there is the lack dataset of the walking ground wire.

The positioning accuracy of the scanner is 0.02 m. The vertical accuracy is 0.068 m and the horizontal accuracy is 0.045 m, which shows the suitability of the acquired data for inspection. There are some discrete noise points above the testing site (purple points), where the quantity and density of the noise points are low. The primary error source was multipath. Black “hole” was labeled as a red circle in Figure 8b, derived from the start position of CIR. The first dataset mainly includes four categories of objects: power line, building, tree, and roof. They can simulate inspection objects in SFD partition, i.e., crossing power line, roof, and tree crown.

### 5.2. Second Dataset

**Figure 9:**
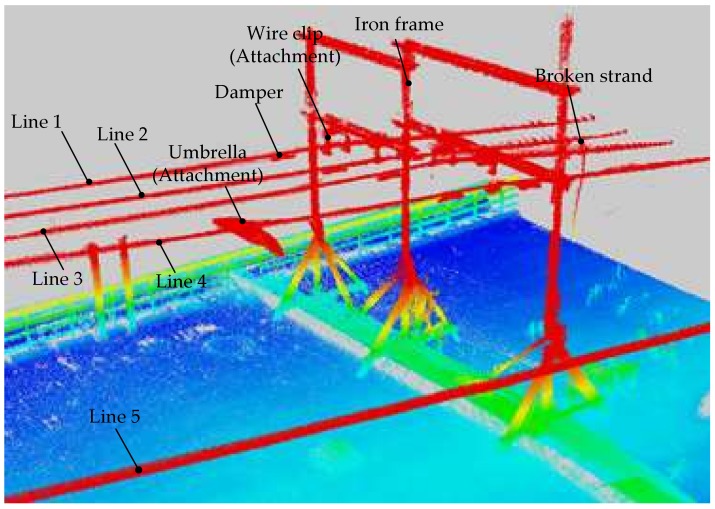
Point cloud of the second dataset.

The testing site was specially constructed to simulate various inspection objects that possibly appear in SPL partition. Four parallel LGJ-95 power lines (Lines 1–4) were installed on two iron frames. Each power line had two dampers. A segment of cable was suspended on Line 2 to simulate a broken strand. An opened umbrella was placed on Line 4 to simulate an attachment. The four wire clips used to support power lines were also considered as attachments. The CIR moved at a speed of 4 km/h along the Line 5 parallel to other lines. Figure 9 shows a more precise dataset, which clearly displays these inspection objects.

### 5.3. Third Datase

**Figure 10:**
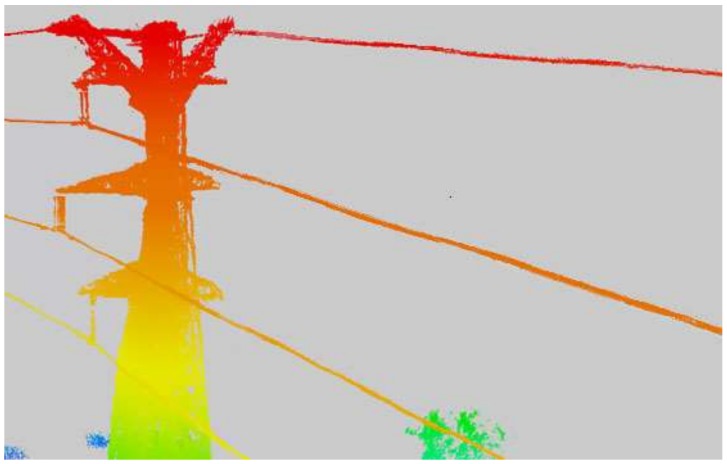
Point cloud of the third dataset.

The third dataset was captured from actual line loaded 220 KV high voltages. The actual line is located in the Changbai Mountains, Jilin Province, China. The area has dense vegetation and undulating terrain. There are five power lines on the tower: two ground wires at the top layer and three-phase power lines under the same side of the walking ground wire. The third dataset was used to verify the feasibility of the proposed method. Figure 10 is the rendered image of third dataset by elevation in which the walking ground wire was missing. The inspection objects in the third dataset mainly include a damper, insulator, and tree crown. There are no other inspection objects (e.g., attachment, broken strand) in the actual line with the naked eye.

**Figure 11:**
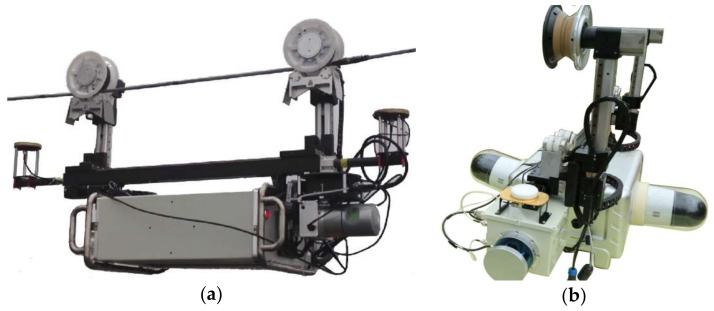
The two CIR LiDAR systems: (**a**) System 1; (**b**) System 2.

The three datasets were collected from two CIR LiDAR systems. The first and third datasets were collected by System 1, as shown in Figure 11a. A laser scanner was directly installed below the antenna, leading to blocking the upward scanning angle of LS, so that there was only a power line in the first dataset in Figure 8. The third dataset also misses the walking ground wire (in Figure 10). The second dataset was collected by System 2 in Figure 11b. The upward scanning angle of LS is not covered, and the installation size is beyond the minimum scanning distance of LS (1 m), so the dataset contains all power lines of the testing site (in Figure 9).

Table 2 summarizes the specifications of the three datasets in terms of the two LiDAR systems, the number of points, and the densities and dimensions of the covered areas. The three datasets can show that the magnitude of CIR LiDAR datasets is large, for a span segment of a point cloud over 10^7^. The point cloud density is more than three orders of magnitude of the ALS point cloud, providing a strong foundation for precise inspection object recognition.

The first dataset only has anSFD partition. The second dataset was divided into four sub-partitions (i.e., *SPL*_1_, *SPL*_2_, *SPL*_3_, *SPL*_4_). The third dataset has three sub-partitions (*SPL*_1_, *SPL*_3_, *SPL*_5_, sorting by the partition rules.) and an SFD partition. The distributions of inspection objects for the three datasets are shown in Table 3. In SPL partition, the numbers of inspection objects are listed in sequence according to the four categories: damper, insulator, broken strand, and attachment. In SFD partition, the numbers of inspection objects are listed in sequence according to three categories: power line, roof, and tree crown. The total number of inspection objects is listed in the last column of Table 3.

## 6. Results and Discussion

### 6.1. Analysis of Ground Point Removal

Table 4 presents the results of ground point removal in the three datasets. From these datasets, it can be seen that the number of original point clouds of CIR LiDAR is large, and the ground points with an average of 79.7% of the dataset can be quickly removed by ground point removal. Among them, the ground point of the third dataset is only 50.3%, because it contains a lot of tower points. We do not consider point clouds of the tower, so the average ground point percentage of the three datasets can reach above 95%. Furthermore, the numbers of inspection object points extracted by mistake are zero, which shows that the proposed method can not only effectively remove ground points in datasets, but also retain all meaningful point clouds closely associated with power lines in a span segment.

### 6.2. Analysis of Power Line Partition

Table 5 shows the statistics results of power line partition in three datasets. The number of each partition point is reduced to five orders of magnitude, and accounts for an average of 0.57% of the total dataset. Among them, the SFD partition percentage of the first dataset is 2.7%, which is obviously higher than the other partitions, because the partition contains a dense tree crown and a large area of the roof. The other partitions account for an average of only 0.31% of their corresponding datasets. Obviously, the partition strategy from “layer” to “block” is beneficial to data classification and computational efficiency. The omission number represents the extraction quality of each partition. The results show that the proposed method has a good extraction effect in the third dataset. Only in the second dataset, the segment of cable to simulate the broken strand is missing part of the line segment. The reason for this is that the testing site limited by size is not strictly constructed according to the erection specification. The high-quality partition lays a good foundation for the following inspection object recognition.

### 6.3. Analysis of SPL Partition Recognition

**Figure 12:**
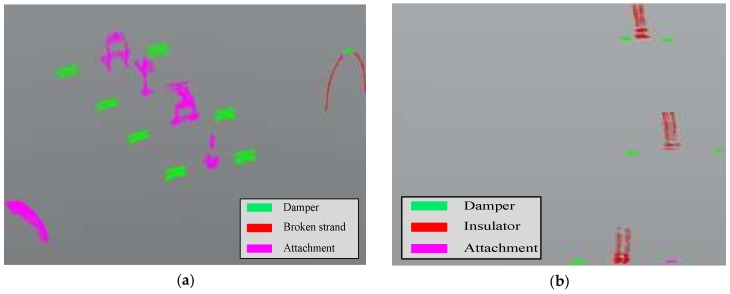
Object recognition of the second dataset (**a**), the third dataset (**b**) in SPL partition.

In SPL partition, the inspection objects are divided into four categories: damper, insulator, broken strand, and attachment, which are characterized by a small size and complicated shape. Figure 12 shows the recognition results of the proposed method in SPL partition. There are eight dampers, one broken strand, and five attachments in the second dataset. Among them, seven dampers were correctly identified and one damper was misjudged as an attachment; most of the broken strands were correctly identified, although a small part of it was misjudged as a damper and attachment; the attachments were correctly recognized, as shown in Figure 12a. There are six dampers and three insulators in the third dataset. It can be seen in Figure 12b that five dampers were correctly recognized, one damper was misjudged as the attachment; three insulators were correctly identified, but parts of the point clouds of the lowest insulator were misjudged as the attachment.

**Figure 13:**
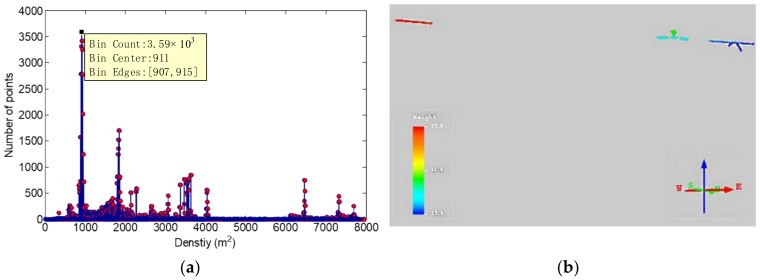
Density histogram of a partition in the second dataset (**a**) and corresponding extraction results (**b**).

Compared with Figure 12 and the original point clouds of the datasets in Figure 9 and Figure 10, the sizes of inspection objects in SPL partition are small, especially in the actual line. Therefore, the removal of power line points in a sub-partition can reduce the interference of power line points and highlight the characteristics of inspection objects. Figure 13 shows the result of removing power line points in a sub-partition for the second dataset. Figure 13a is the density distribution of a sub-partition. The peak value of the density histogram was used as the extraction threshold to obtain the abnormal point clouds, as shown in Figure 13b. These point clouds include all inspection objects in the sub-partition. Because they have no power line point connection, they present the “block” distribution, reducing mutual interferences of inspection objects data. It is beneficial to the classifications and recognitions of inspection objects. There was a small number of residual power line points in the point cloud, which can be further removed by its linear characteristics.

**Figure 14:**
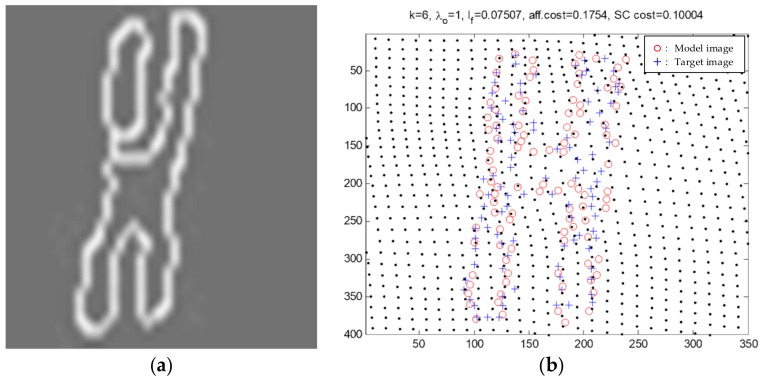
Edge image of a damper in the third dataset by canny algorithm (**a**) and the sixth iteration result by the SC algorithm (**b**).

The fittings in SPL partition are the main inspection objects, and they usually have complex geometric shapes. The proposed method combines their distinctive topologies with the image processing method to obtain a good recognition result. As an example, a damper of the third dataset was used to illustrate the process of artificial facility recognition in Figure 14. Waiting-recognition points are used to generate the edge image through preprocessing of the Canny algorithm. It can be clearly seen from Figure 14a that the edge image is the contour of a damper. Figure 14b indicates the sixth iteration result of SC matching for the image. Figure 14 shows that CIR LiDAR can obtain the precise point cloud of a small-size inspection object, which is the data foundation of the proposed method.

The results assessment are presented in Table 6 in terms of the recognition accuracy and precision and recall at the point cloud level. The three indexes are calculated as follows:

(8) $$\begin{array}{l} accuracy=\frac{tp+tn}{tp+tn+fp+fn}\\ precision=\frac{tp}{tp+fp}\\ recall=\frac{tp}{tp+fn} \end{array},$$

Compared with Table 6 and Figure 12a, for the second dataset, the recognition precision of damper was 95.3%, and the reason for the misjudgment was that the shape of the crossed position of the cable and power line was similar to the shape of the damper. The recognition recall of the damper was 94.4%. The reason for this is that the damper was in the dense area (iron frames and cable clamps) in which the incomplete contour induced by occlusion was a misidentified attachment. Therefore, the recognition accuracy of the damper was 89.7%. There was no insulator in the testing site. For the broken strand, its recognition accuracy was only 52.5%, because parts of the crossing power line points were misidentified as a damper and attachment. But the broken strand was not misidentified, with a recognition precision of 100%. All attachments in the partition were correctly recognized, so the recall rate was 100% and the recognition accuracy of attachment was 92.5%.

According to Table 6 and Figure 12b, in the third dataset, the recognition accuracy of the damper was 87.4%. Part of the missing point clouds of dampers blocked by the tower facilities was misidentified as an attachment. The recognition precision of the insulator was 100%. Only a small number of points were misidentified as an attachment due to occlusion, so its recognition accuracy was better than 98%.

### 6.4. Analysis of SFD Partition Recognition

**Figure 15:**
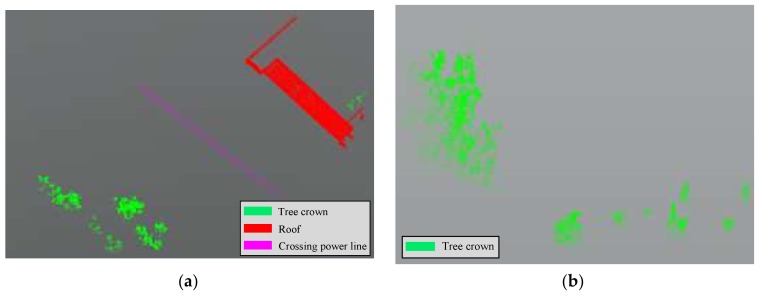
Recognized objects of the first dataset (**a**), the third dataset (**b**) in SFD partition.

In SFD partition, the inspection objects are divided into three categories: crossing power line, roof, and tree-crown. Figure 15 shows the recognition results of the proposed method in SFD partition. The inspection objects in SFD partition are generally large sizes and simple categories, especially in the actual line, so they can be well recognized using the 3D shape analysis and region growing.

Compared with Table 7 and Figure 15, in the first dataset, the testing site has one crossing power line correctly recognized without misjudgment or omission. The first dataset was collected with System 1, which has twice as much laser head as System 2, so the point cloud density was greater, and the accuracy of the point cloud was also better. Moreover, there was no fitting or attachment installed on the power line, and the linear characteristics were obvious. The recognition accuracy of the roof was 98.8%. Only part of these points was covered during scanning, and the missing data was misidentified as a tree crown. The tree crown in the partition was correctly identified and the recall rate was 100%. In the third dataset, the object in the actual line was relatively simple, with only a few tree crowns which were correctly recognized.

As the results suggest, under-sampling, caused by coverage or occlusions, is the primary cause of detection failure or false positives, especially for the small-size objects such as dampers. When the number of power lines is large, occlusions are more likely to happen. Their shapes and structures cannot be recognized according to the description rules. We can use CIR LiDAR to acquire more collections on the two ground wires to ameliorate this situation. This action, however, requires additional time for fieldwork and results in a span segment of a point cloud with a larger volume, which increases computational time and cost and decreases data processing efficiency. Therefore, only when there are more power lines, occlusions are more serious, or it is desirable to obtain higher recognition results, would it be possible to consider this correction method. Moreover, sudden changes in the external environment, such as strong wind, could cause errors in the calculation of the additional sag. This may affect the partition results, causing recognition failure and false positives. We can optimize the information of sensors on CIR’s body to ameliorate this situation.

## 7. Conclusions

In this study, a new method is proposed to recognize inspection objects surrounding power lines or around the corridors in support of improving the defense capability of the power grid. CIR LiDAR data is sufficiently dense. The number of single-span point clouds is more than 10^7^. Point cloud density is more than three orders of magnitude relative to the ALS point cloud, obtaining precise topologies of small-size inspection objects. The optimal elevation threshold constructed by the CIR POS can be used to quickly remove more than 95% of ground points. The extraction model is constructed by the SPPD algorithm based on CIR POS. Point cloud partition from “layer” to “block” is fully combined with the characteristics of working conditions. The advantage of the SPPD algorithm is that it is able to extract the data of a single power line and its surrounding completely following the shapes of the actual lines. The recognition works are fully focused on a single power line, so as to solve the problem of small-size inspection objects being difficult to directly extract from the original point clouds. Finally, we propose a partition recognition method that defines SFD and SPL partitions to solve the recognition problems caused by the great-size differences of inspection objects. The local neighborhood statistics and the 3D region growing method are used for the recognition of inspection objects in the two partitions. The three datasets achieved an average accuracy of 95.6% at the object level, and achieved an average accuracy of 90.6% and an average precision of 98.2% at the point cloud level, showing a feasible and promising method to extract inspection objects of power lines.

Further research works should mainly focus on two aspects: One is that when CIR LiDAR collects data along the ground wire, the external environment could introduce high noise, such as strong wind, robot skid, etc. How to guarantee the performance of the proposed method can be studied in such conditions. The other is the classification and recognition methods of point clouds, which should be further updated and developed. In the future, focusing on the categories and features of inspection objects, the recognition algorithm will be further improved in terms of the detection efficiency and recognition accuracy.

## Figures and Tables

**Table 1:** Characteristic rules of inspection objects.

Inspection Object	Feature Description	Density	Contour Feature	Normal Vector
Damper	■ Artificial facility; ■ Attached below the power line; ■ Shape specification.	Dense	Cylinder shape	Horizontal direction
Insulator	■ Artificial facility; ■ Attached above the power line; ■ Shape specification.	Dense	Cylinder shape	Vertical direction
Broken strand	■ Attached the power line.	Denser	Curve shape	Varied direction
Attachment	■ Irregular shape; ■ Small area; ■ Attached on the power line.	Dense	Irregular shape	Varied direction
Tree crown	■ Irregular shaped; ■ Big area; ■ Below all power lines.	Dense	Irregular shape	Varied direction
Overrun building	■ Irregular rectangle; ■ Big area; ■ Below all power lines.	Dense	Rectangle	Vertical direction
Crossing power line	■ Below all power lines.	Ordinary	Linear	Horizontal direction

**Table 2:** Specifications of three datasets used in the paper.

Specifications	1st Dataset	2nd Dataset	3rd Dataset
Laser scanner	Velodyne 32E	Velodyne VLP16	Velodyne 32E
POS	SPN-IGM-A1	APX-15UAV	SPN-IGM-A1
Number of points	26,389,079	24,633,871	69,969,137
Density(/m^2^)	6584	5139	5936
Area(m^2^)	143 × 79	174 × 97	343 × 148

**Table 3:** Object distributions of the three datasets.

Dataset	*SPL*_1_	*SPL*_2_	*SPL*_3_	*SPL*_4_	*SPL*_5_	SFD	Sum
	(Damper, Insulator, Broken Strand, Attachment)					(Power Line, Roof, Crown)	
1st Dataset	-	-	-	-		(1,1,4)	6
2nd Dataset	(2,0,0,1)	(2,0,1,1)	(2,0,0,1)	(2,0,0,2)		-	14
3rd Dataset	(2,1,0,0)	-	(2,1,0,0)	-	(2,1,0,0)	(0,0,8)	17

**Table 4:** The results of ground point removal in the three datasets.

Dataset	Totality	Ground Points	Percentage	Object Points
1st Dataset	26,389,079	25,673,711	97.3%	0
2nd Dataset	24,633,871	22,511,542	91.4%	0
3rd Dataset	69,969,137	35,199,149	50.3%	0

**Table 5:** The statistics results of point clouds in different partitions for three datasets.

Dataset	1st Dataset	2nd Dataset				3rd Dataset			
Partition	SFD	*SPL*_1_	*SPL*_2_	*SPL*_3_	*SPL*_4_	*SPL*_1_	*SPL*_3_	*SPL*_5_	SFD
Point number	715,368	77,897	93,745	69,739	106,972	139,931	129,840	129,336	335,003
Percentage	2.7%	0.32%	0.38%	0.28%	0.43%	0.20%	0.19%	0.18%	0.48%
Omission number	0	0	569	0	0	0	0	0	0

**Table 6:** Object recognition at point cloud level in SPL partition (in percentage).

**2nd Dataset**	**Damper**	**Insulator**	**Broken Strand**	**Attachment**	**Accuracy**	**Precision**	**Recall**
Damper	14,103	0	0	841	89.7	95.3	94.4
Insulator	0	0	0	0	-	-	-
Broken strand	698	0	3031	2046	52.5	100	52.5
Attachment	0	0	0	35,466	92.5	92.5	100
**3rd Dataset**	**Damper**	**Insulator**	**Broken Strand**	**Attachment**	**Accuracy**	**Precision**	**Recall**
Damper	9812	0	0	1412	87.4	100	87.4
Insulator	0	22910	0	318	98.6	100	98.6
Broken strand	0	0	0	0	-	-	-
Attachment	0	0	0	0	0	0	-

**Table 7:** Object recognition at point cloud level in SFD partition (in percentage).

**1st Dataset**	**Crossing Power Line**	**Roof**	**Tree Crown**	**Accuracy**	**Precision**	**Recall**
Crossing power line	33,557	0	0	100	100	100
Roof	0	515,581	6054	98.8	100	98.8
Tree crown	0	0	143,886	95.8	96.0	100
**3rd Dataset**	**Crossing Power Line**	**Roof**	**Tree Crown**	**Accuracy**	**Precision**	**Recall**
Crossing power line	0	0	0	-	-	-
Roof	0	0	0	-	-	-
Tree crown	0	0	335,003	100	100	100
